# Evaluation of the healing potential of short-term ozone therapy for the treatment of diabetic foot ulcers

**DOI:** 10.3389/fendo.2023.1304034

**Published:** 2024-01-15

**Authors:** Haojie Sun, Hao Heng, Xuekui Liu, Houfa Geng, Jun Liang

**Affiliations:** Xuzhou Central Hospital, Xuzhou Clinical School of Xuzhou Medical University, Xuzhou, China

**Keywords:** diabetic foot ulcers, ozone therapy, short-term, wound, healing

## Abstract

**Background:**

The availability of research on short-term ozone therapy for diabetic foot ulcers (DFUs) is limited, and even when it is accessible, it mainly comprises of basic analysis conducted during long-term ozone therapy. This study was to evaluate the efficacy of short-term ozone therapy in promoting wound healing in DFUs.

**Methods:**

A retrospective analysis was conducted on 89 patients with type 2 diabetes complicated by DFUs. The patients were divided into two groups: ozone therapy group (n=41) and control group (n=48). Wound condition, change of bacterial types, changes in inflammatory indicators (erythrocyte sedimentation rate [ESR], C-reactive protein [CRP], and procalcitonin [PCT]), vascular endothelial growth factor (VEGF), cytokines [Interleukin 6 (IL-6) and tumor necrosis factor-α(TNF-α)], and oxidative stress levels (superoxide dismutase [SOD], malondialdehyde [MDA], and total antioxidant capacity [T-AOC]) were observed pre-treatment and after 1 week. After a 12-week of follow-up, wound healing rate, amputation rate, inpatient day, duration of antibiotics, reinfection rate, incidence of new ulcers, readmission rate, and reoperation rate, and cumulative wound healing rate using Kaplan-Meier curves were assessed.

**Results:**

After 1 week of treatment, the ozone therapy group showed higher VEGF, SOD, and T-AOC levels compared to the control group (*P*<0.05), while CRP, PCT, ESR, IL-6, TNF-α, MDA levels and bacterial types were lower (*P*<0.05). The ozone therapy group had a higher wound healing rate after a 12-week follow-up (*P*<0.05). Kaplan-Meier curves indicated a higher cumulative wound healing rate in the ozone therapy group (*P*<0.05). Additionally, the ozone therapy group had lower inpatient day, duration of antibiotics, reinfection rate, and readmission rate compared to the control group (*P*<0.05).

**Conclusion:**

Short-term ozone therapy is effective in promoting wound healing in DFUs by reducing inflammation, increasing growth factor levels, improving oxidative stress status, shortening healing time, and improving long-term prognosis. These findings suggest the potential of short-term ozone therapy as a valuable treatment modality for DFUs.

## Introduction

Diabetic foot ulcers (DFUs) is a severe complication of diabetes that can result in ulcers, infections, or tissue damage in the feet. By 2025, it is estimated that over 125 million out of 500 million people with diabetes globally will develop foot ulcers (1). The prevalence of DFUs in the global population is 6.3% (2). Shockingly, every 20 seconds, one patient loses a leg due to diabetes (3). The annual mortality rate among DFUs patients is as high as 11%, while amputees face a staggering 22% rate (4). DFUs treatment is challenging and expensive, burdening patients and their families both psychologically and economically, and posing significant challenges for healthcare systems worldwide.

DFUs is complex and often accompanied by an imbalance in oxidative stress. Hyperglycemic DFUs patients experience an accumulation of excessive peroxides in their bodies. This oxidative stress accelerates cell apoptosis, damages the microvasculature, and hinders DFUs wound healing. Thus, oxidative stress plays a crucial role in the development and control of DFUs (5).

Traditional wound dressing methods frequently result in chronic non-healing wounds, adding to the difficulty and cost of clinical treatment. Ozone, composed of three oxygen atoms, rapidly breaks down into oxygen, with one oxygen atom serving as a potent oxidant that can eliminate microorganisms and activate antioxidant enzymes (6). Studies have demonstrated the application of ozone therapy in various conditions, including periodontitis, pain management, tumors, and diabetic wounds (7–10). As an adjunct therapy for DFUs, the effectiveness of ozone therapy varies. Some research suggests that ozone therapy is more effective than standard treatment for DFUs management (11). However, there is also a study indicating that ozone therapy has no significant impact on DFUs healing (12). The duration of ozone therapy for DFUs varies among studies, with treatments ranging from a 12-14 day therapy performed by Rosul et al. to a continuous 20-day therapy conducted by Zhang et al. (13, 14). The frequency of ozone therapy is generally once daily, twice weekly, or once every three days. (12, 14, 15). Hospitalized patients can typically adhere to this therapy frequency, but for discharged patients living far from medical facilities, maintaining the prescribed ozone therapy frequency can present challenges, leading to potential treatment interruptions. Research on short-term ozone therapy for DFUs is scarce, and even if available, it primarily consists of basic analysis conducted during long-term ozone therapy. Given these considerations, we propose investigating the effectiveness of short-term ozone therapy for DFUs. Thus, this study aims to evaluate the short and long period efficacy of short-term ozone therapy in hospitalized DFUs patients and provide valuable clinical guidance for the management of DFUs using ozone therapy.

## Materials and methods

### Study subjects

A retrospective analysis was conducted on 89 hospitalized patients with type 2 diabetes complicated by DFUs between July 2022 and April 2023. Of the participants, 55 were males and 34 were females, with ages ranging from 40 to 74 years (62.89± 7.64). The duration of diabetes ranged from 1 to 23 years (10.46 ± 5.52), while the duration of DFUs ranged from 1 week to 48 weeks (6.31 ± 6.93). DFUs diagnosis followed the diagnostic criteria set by the International Diabetic Foot Working Group Guidelines (16). Participants were divided into two groups based on treatment method: the ozone therapy group (n=41) and the control group (n=48). The ozone group received ozone therapy in addition to the treatment provided to the control group.

Inclusion criteria were as follows: (1) age between 18 and 80 years; (2) ankle-brachial index between 0.7 and 1.2; (3) wound area >4cm^2^; (4) Wagner grade 2, 3, or 4.
Exclusion criteria included: (1) patients with malignant transformation of diabetic ulcers; (2) patients with severe primary diseases affecting the heart, brain, liver, kidney, hematopoietic system, or mental health; (3) patients who did not adhere to prescribed treatment or had incomplete clinical data that could affect evaluation of treatment efficacy; (4) patients intolerant to treatment and experienced adverse reactions; (5) patients with connective tissue diseases; (6) active Charcot foot syndrome; (7) other infectious or contagious diseases.
Contraindications: (1) Glucose-6-phosphate dehydrogenase deficiency; (2) Toxic thyroid hyperfunction; (3) Platelet count below 50,000 and severe coagulation disorders; (4) Acute alcohol poisoning; (5) Excessive and acute bleeding; (6) Seizure condition; (7) Hemochromatosis; (8)Patients undergoing copper or iron therapy.

The study protocol was explained to participants, and written informed consent was obtained. The study was approved by the Ethics Committee of Xuzhou Central Hospital (XZXY-LK-20220629-055) and conducted in accordance with the Helsinki Declaration.

### Data collection

Clinical data collection included demographic information, diabetes complications and comorbidities, duration of diabetes, wound location, and wound severity.

### Basic treatment

Individualized treatment approaches were provided based on patients’ conditions, including glycemic control, improved circulation, infection control, nutritional support, blood pressure management, lipid control, and offloading strategies. Antibiotics were empirically administered in the early stages based on clinical judgment. Then, based on the culture of wound necrotic tissue microorganisms, susceptibility testing, and clinical response, antibiotics were adjusted. Toe amputation and debridement were performed based on foot conditions, targeting osteomyelitis and necrotic bones.

### Ozone therapy

**Figure 1:**
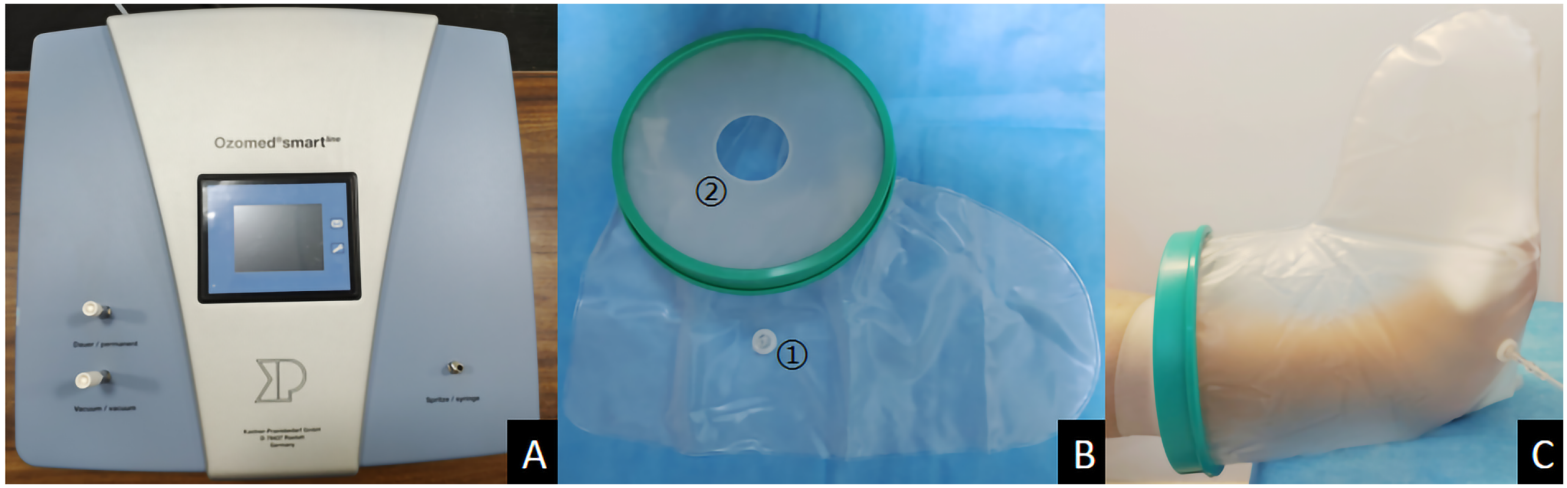
Photo illustrations ozone therapy for diabetic foot ulcer. **(A)** Kastner Ozomed Smart Devices; **(B)** Ozone bag:①Ozone ventilation port ②Sealing ring (high elasticity and good sealing performance); **(C)** Ozone therapy for diabetic foot ulcer.

**Figure 2:**
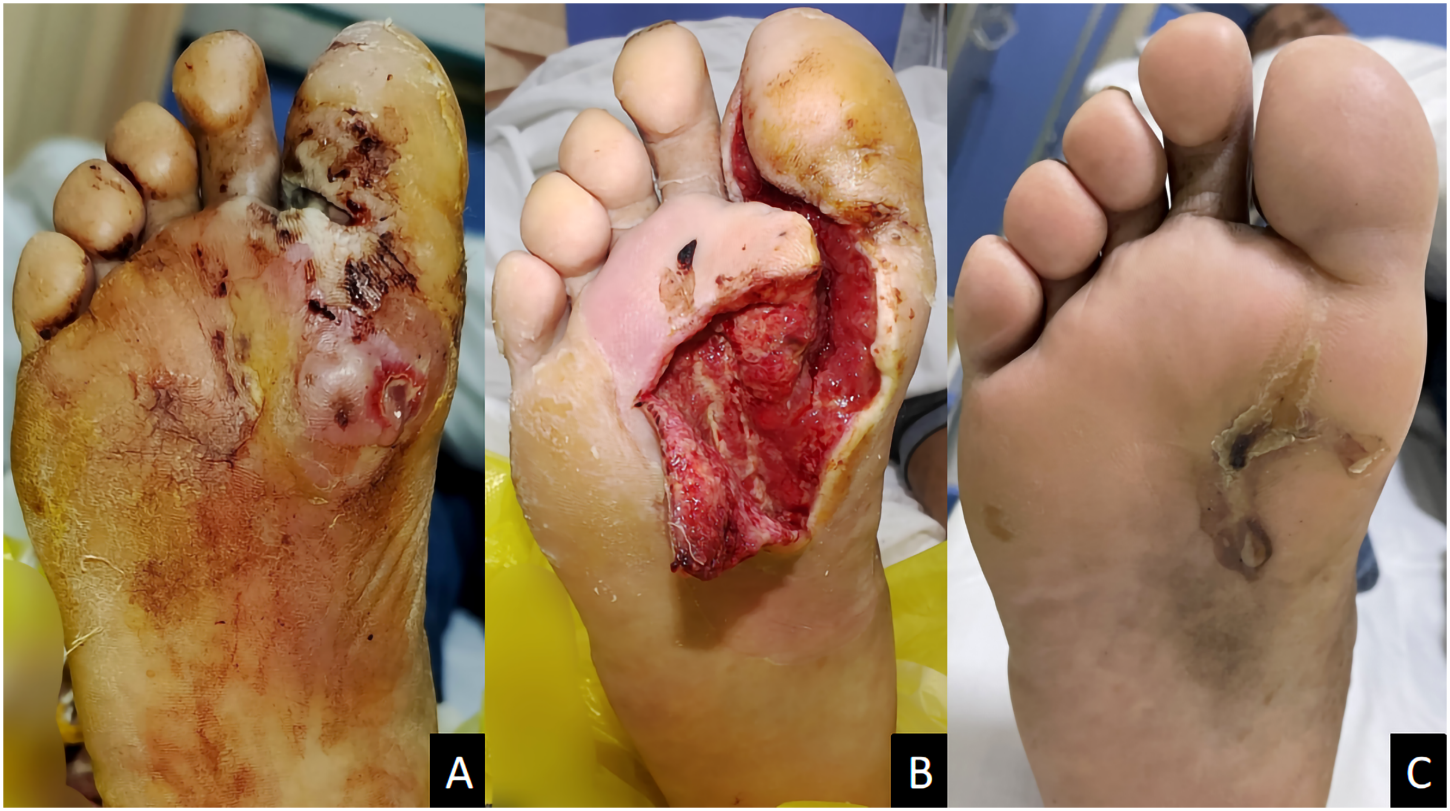
A male diabetic foot ulcer patient aged 64 years with deep abscess on the right foot for 2 weeks. **(A)** Before treatment; **(B)** 1 week after ozone therapy; **(C)** 58 days after treatment.

Ozone therapy was administered using the Kastner Ozomed Smart Devices made in Germany. Patients were comfortably positioned, and wound dressings were opened. Necrotic tissue was debrided. Wound exudate was gently removed using sterile gauze, and the wound was rinsed with 0.9% saline. A disposable plastic tube was placed approximately 2cm away from the wound and secured with adhesive tape. The other end of the tube was connected to the vacuum hole of the ozone therapy instrument. A sterile ozone bag was applied to cover and seal the limb (The sealing ring is pulled apart, slowly sliding the affected foot into the ozone to avoid touching the wound. Once the wound is fully covered, the sealing ring is adjusted for a tight fit.). The air in the bag was evacuated, and medical ozone gas (ozone concentration of 35 μg/ml) was introduced into the bag for 30 minutes. (**Figure 1**) Therapy was administered once a day, with close monitoring of patient condition. The plastic bag should be securely sealed to prevent any air leakage. Additionally, it is crucial to maintain a steady room temperature and ensure good indoor ventilation. Ozone therapy was immediately stopped in case of adverse reactions. At the end of treatment, ozone was withdrawn and decomposed from the bag through the Kastner Ozomed Smart Devices. The bag was then separated from the patient, and the wound was covered and dressed with sterile dressings. (**Figure 2**) All patients underwent conventional dressing changes using sterile Vaseline gauze.

### The Bates-Jensen wound assessment tool

The Bates-Jensen wound assessment tool, consisting of 13 wound characteristics, was used to assess the wounds (17). Each characteristic had five levels of description. Scores were assigned to each level, and the total score was obtained by summing all individual item scores. A higher total score indicated a more severe wound condition. After 1week of treatment, wound scores were evaluated.

### Blood specimen collection

Peripheral venous blood samples were collected from patients in both groups in the morning, after overnight fasting, at the baseline and after 1 week of treatment.

### Endpoint

The primary outcome was the rate of complete wound closure at 12 weeks. Complete wound closure was defined as full epithelialization without any breakdown or exudate. Secondary outcomes included index hospitalization outcomes (surgeries during admission and inpatient day), outcomes after hospital discharge (duration of antibiotics, healed at end of study, new ulcer formation rate, reinfection rate, readmission rate, surgery after discharge), change of bacterial types, and wound scores, changes in inflammatory markers (ESR, CRP, and PCT), VEGF, IL-6, TNF-α, and oxidative stress levels (SOD, MDA, and T-AOC) in serum before and after 1 week of treatment.

### Statistical methods

Data analysis and graph plotting were performed using SPSS version 21.0 and GraphPad Prism version 9. Differences in normally distributed variables were analyzed Student’s t-test. Non-normally distributed variables were analyzed using non-parametric tests. Count data is typically presented as frequencies (in percentages) and analyzed using the chi-square test. Numeric variables were tested using the Kaplan-Meier (K-M) test to assess the distribution. All tests were two-sided, and a significance level of 0.05 was set.

## Results

### Comparison of baseline characteristics

There were no significant differences in baseline characteristics between the ozone therapy group and the control group (all *P*>0.05), as shown in **Table 1**.

**Table 1:** Comparison of clinical characteristics between the two groups.

Variables	Group		P-value
	Control	Ozone	
N	48	41	
Gender (male/female)	30/18	25/16	0.883
Age (years)	62.73 ± 6.78	63.07 ± 8.63	0.609
Smoking (%)	19 (39.6)	14 (34.1)	0.597
Drinking (%)	16 (33.3)	17 (41.5)	0.429
Duration of Diabetes (year)	10.54 ± 5.70	10.37 ± 5.38	0.755
Duration of Diabetic Foot Ulcers (weeks)	6.54 ± 7.77	6.05 ± 5.87	0.967
FPG (mmol/L)	8.97 ± 2.82	10.40 ± 4.03	0.124
HbA1c (%)	9.95 ± 2.08	10.60 ± 2.92	0.241
Albumin (g/dL)	35.37 ± 4.98	34.46 ± 4.94	0.378
eGFR (mL/min/1.73m2)	95.72 ± 16.07	94.86 ± 15.41	0.798
Wound Area (cm^2^)	11.93 ± 3.59	12.82 ± 3.93	0.251
ABI	0.84 ± 0.11	0.82 ± 0.12	0.248
Hypertension (%)	16 (33.3)	15 (36.6)	0.748
Coronary Artery Disease (%)	14 (29.2)	12 (29.3)	0.992
Cerebral Infarction (%)	11 (20.8)	9 (22.0)	0.913
Diabetic Retinopathy (%)	27 (56.3)	21 (51.2)	0.635
Diabetic Peripheral Neuropathy (%)	30 (62.5)	27 (65.9)	0.742
Wagner Grade (%)			0.971
2	10 (20.8)	8 (19.5)	
3	25 (52.1)	21 (51.2)
4	13 (27.1)	12 (29.3)
Wound Location			0.937
Forefoot	36 (75.0)	32 (78.0)	
Midfoot	7 (14.6)	5 (12.2)	
Heel	5 (10.4)	4 (9.8)	

Continuous variables are expressed as Mean ± SD.

FPG, fasting plasma glucose; HbA1c, glycosylated hemoglobin; eGFR, estimated glomerular filtration rate; ABI, ankle-brachial index.

### Comparison of Bates-Jensen wound assessment tool scores

After 1 week of treatment, the ozone therapy group showed significantly lower wound scores compared to the control group (*P*<0.001) (**Table 2**).

**Table 2:** Comparison of Bates-Jensen wound assessment tool score between the two groups.

	Control	Ozone	p
Before-treatment	47.42 ± 3.55	48.07 ± 3.35	0.374
After-treatment	44.15 ± 3.94	40.37 ± 3.69	<0.001

### Comparison of inflammatory markers

**Figure 3:**
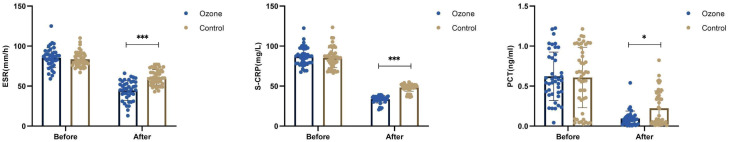
Comparison of the inflammatory markers between the two groups. "*" <0.05; "***" <0.001.

There were no differences in ESR (83.58 ± 9.05 vs 85.41 ± 12.53 mm/h, *P*=0.427), CRP (85.32 ± 12.35 vs 87.13 ± 11.51mg/L, *P*=0.476), and PCT (0.61 ± 0.38 vs 0.62 ± 0.30ng/ml, *P*=0.860) levels between the control group and the ozone therapy group before treatment. However, after 1 week of treatment, there were significant differences in ESR (61.04 ± 9.56 vs 44.59 ± 12.47 mm/h, *P*<0.001), CRP (48.17 ± 4.65 vs 33.54 ± 4.58mg/L, *P*<0.001), and PCT (0.22 ± 0.22 vs 0.09 ± 0.09ng/m, *P*<0.05) levels (**Figure 3**).

### Comparison of cytokine levels

**Figure 4:**
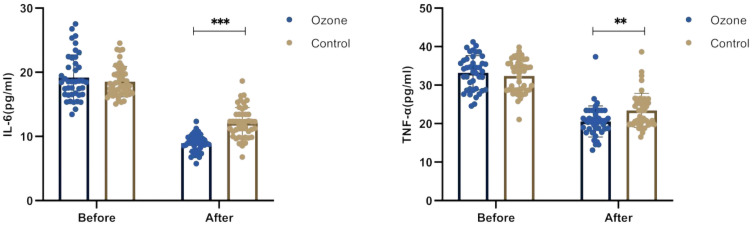
Comparison of the serum cytokine between the two groups. "**" <0.01; "***" <0.001.

Before treatment, there were no differences in IL-6 (18.54 ± 2.36 vs 19.18 ± 3.52 ng/L, *P*=0.607) and TNF-α (32.38 ± 4.27 vs 33.21 ± 4.42 ng/L, *P*=0.494) levels between the control group and the ozone therapy group. However, after 1 week of treatment, there were significant differences in IL-6 (12.10 ± 2.39 vs 8.96 ± 1.37 ng/L, *P*<0.001) and TNF-α (23.42 ± 4.42 vs 20.57 ± 4.04 ng/L, *P*<0.01) levels (**Figure 4**).

### Comparison of growth factors

**Figure 5:**
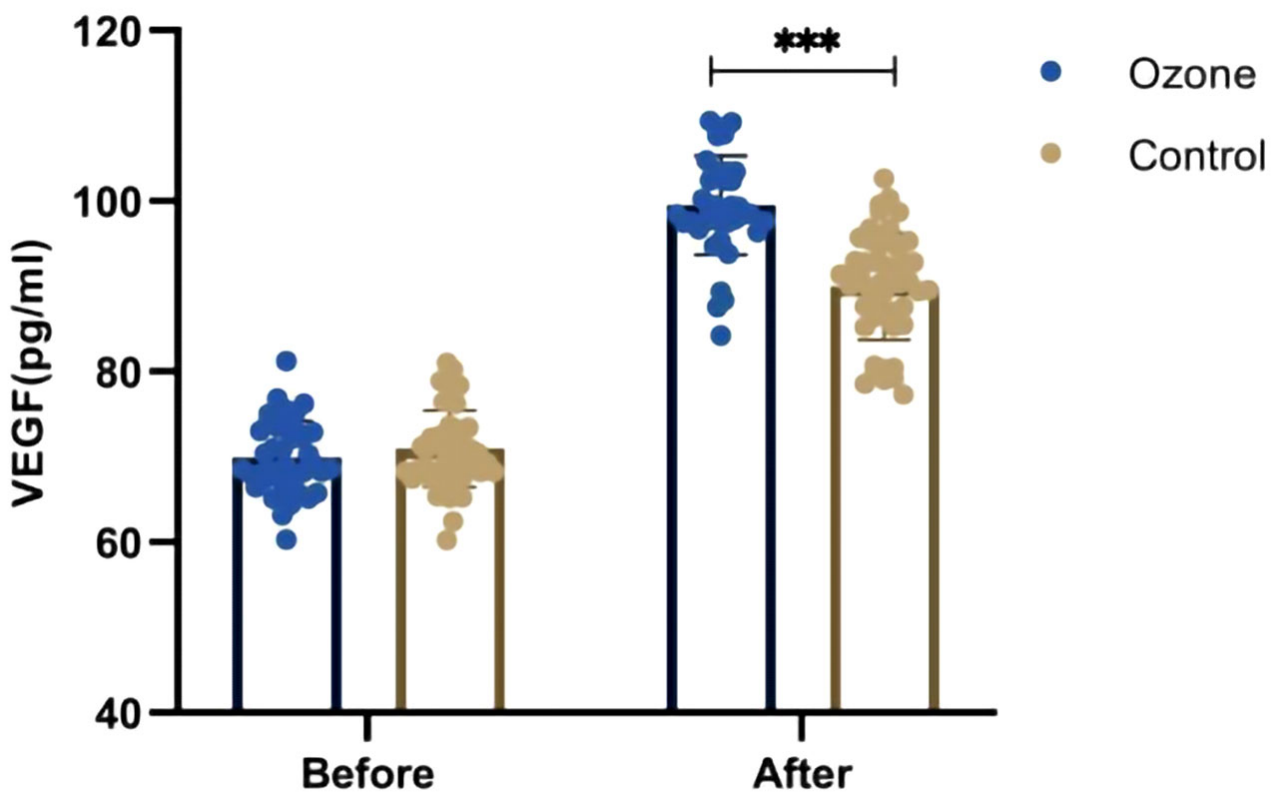
Comparison of the serum VEGF between the two groups. "***" <0.001.

Before treatment, there were no differences in average serum VEGF (70.94 ± 4.49 vs 69.93 ± 4.23 ng/L, *P*=0.281) levels between the control group and the ozone therapy group. However, after 1 week of treatment, there were significant differences in VEGF (89.98 ± 6.26 vs 99.50 ± 5.81 ng/L, *P*<0.001) levels (**Figure 5**).

### Comparison of oxidative stress

**Figure 6:**
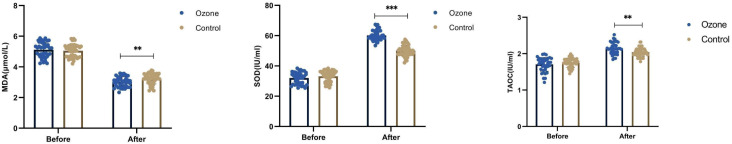
Comparison of the oxidative stress between the two groups. "**" <0.01; "***" <0.001.

Before treatment, there were no differences in SOD (33.18 ± 3.52 vs 32.01 ± 3.91 IU/ml, *P*=0.138), T-AOC (1.75 ± 0.13 vs 1.71 ± 0.19 IU/ml, *P*=0.484), and MDA (5.05 ± 0.41 vs 5.12 ± 0.49μmol/L, *P*=0.376) between the control group and the ozone therapy group. After 1 week of treatment, both groups showed increased SOD (50.06 ± 3.51 vs 60.13 ± 3.42 IU/ml, *P*<0.001) and T-AOC (2.04 ± 0.12 vs 2.14 ± 0.16 IU/ml, *P*<0.01), and decreased MDA (3.23 ± 0.34 vs 3.02 ± 0.33μmol/L, *P*<0.01). The differences were more significant in the ozone therapy group, and all differences were statistically significant (**Figure 6**).

### Change of bacterial types between the two groups

A total of 63 bacterial strains were isolated in the control group, while 55 bacterial strains were isolated in the ozone therapy group. Following 1 week of treatment, the ozone therapy group exhibited a reduced bacterial strains on the wound compared to the control group (2/55[3.6%] vs 9/63 [14.3%], *P*=0.047) (**Supplementary Table 1**).

### Comparison of wound healing rate

**Figure 7:**
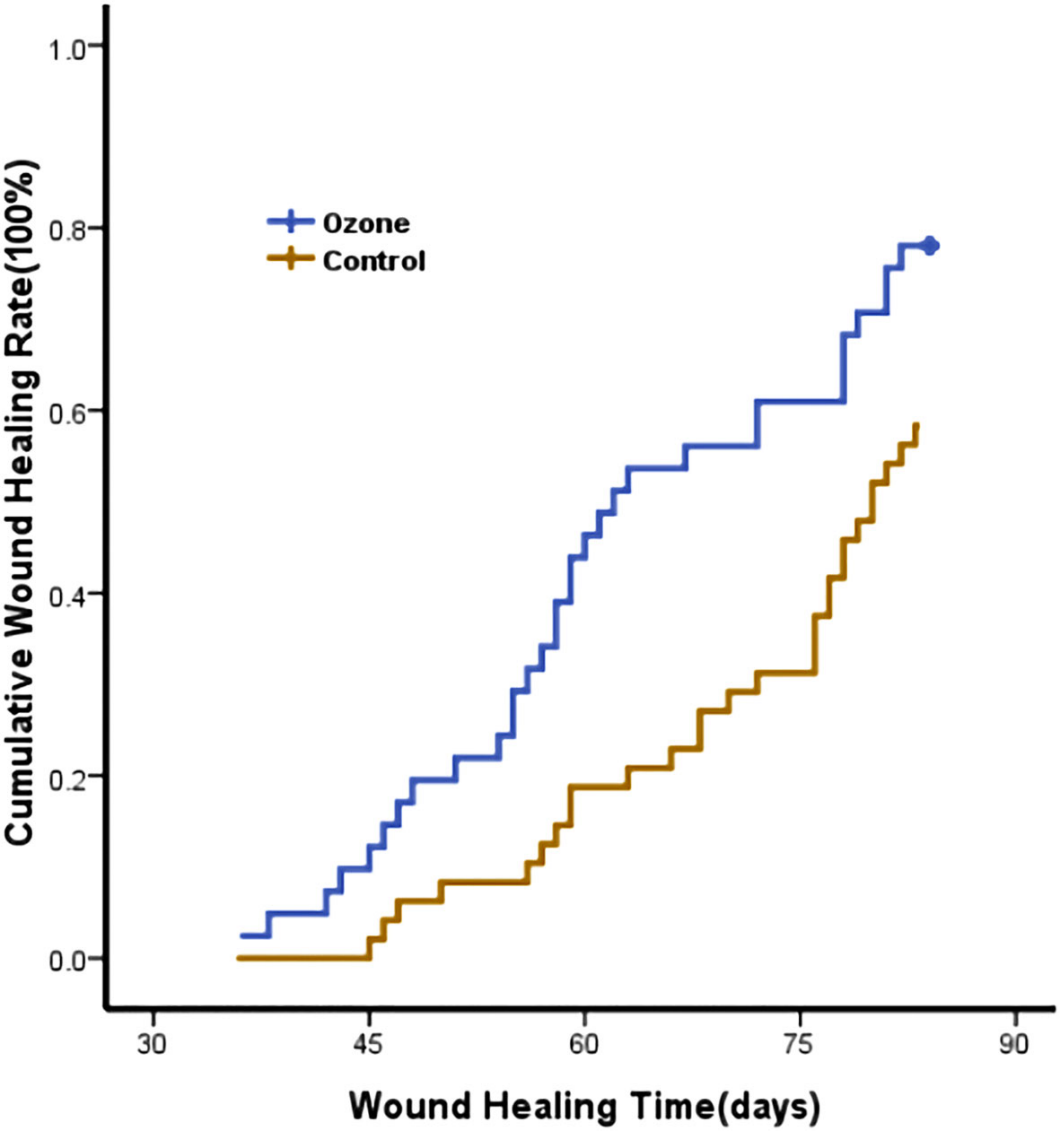
Kaplan-Meier healing curve analysis-days to heal(*p*=0.009).

After a 12-week follow-up, the ozone therapy group had a higher wound healing rate compared to the control group (32/41[78.0%] vs 28/48[58.3%], *P*=0.048), and the cumulative wound healing rate was higher in the ozone therapy group (Log Rank=6.740, *P*=0.009, **Figure 7**). Additionally, the ozone therapy group had shorter inpatient day and duration of antibiotics, and lower rates of reinfection and readmission compared to the control group (*P*<0.05) (**Table 3**).

**Table 3:** Wound Outcomes between the two groups.

Characteristic	Group		P-value
	Control N=48 (%)	Ozone N=41 (%)	
Index Hospitalization Outcomes			
Surgeries during admission			0.980
Debridement	10 (20.8)	8 (19.5)	
Incision, drainage and debridement	19 (39.6)	17 (41.5)
Amputation foot toe and debridement	19 (39.6)	16 (39.0)
Amputation leg and debridement	0 (0.00)	0 (0.00)
Inpatient Day	18.65 ± 4.93	15.66 ± 4.01	0.003
Outcomes After Hospital Discharge			
Duration of Antibiotics (days)	31.42 ± 6.00	27.51 ± 6.03	0.003
Healed at End of Study	28 (58.3)	32 (78.0)	0.048
New Ulcer Formation	8 (16.7)	5 (12.2)	0.552
Reinfection	11 (22.9)	3 (7.3)	0.044
Hospital Readmission Foot	13 (27.1)	4 (9.6)	0.038
Surgery after Discharge	12 (25.0)	4 (9.6)	0.062
Incision, drainage and debridement	6 (50.0)	3 (75.0)	
Amputation foot toe and debridement	6 (50.0)	1 (25.0)	
Amputation leg and debridement	0 (0.00)	0 (0.00)	

### Adverse events

There were no potential human and environmental hazards associated with ozone therapy. Additionally, DFUs patients had no complications or side effects due to ozone therapy.

## Discussion

In this study, the ozone therapy group exhibited a higher healing rate compared to the control group. This finding aligns with a study conducted by Izadi et al., where the ozone therapy group had a greater healing rate than the control group after a follow-up period of 180 days (100% vs 75%) (15). Additionally, Wainstein observed that at week 24, specifically in the per protocol cohort (PP), the proportion of completely closed wounds was notably higher in the ozone therapy group compared to the control group (81% vs 44%) (18). The possible reasons for this could be the impact of ozone on bacterial cell membranes and the activation of the non-specific immune system. Ozone has oxidative properties that can disrupt the bacterial cell membrane by oxidizing phospholipids and lipoproteins, effectively killing bacteria in a short period of time (19). Additionally, ozone indirectly activates the non-specific immune system, leading to processes such as phagocytosis activation and interferon production (6). This immune activation contributes to the elimination of multiple bacteria, reducing the duration of antibiotic treatment and accelerating the healing process of DFUs. In this study, ozone therapy has shown promising results in reducing the bacterial diversity on the surface of DFUs. The finding aligns with previous research on the subject (12). It’s worth mentioning that in this study, the ozone therapy group showed a shorter duration of antibiotics compared to the control group. Ozone also enhances the activity of enzymes such as superoxide dismutase, hydrogen peroxide, and oxidized glutathione reductase. By doing so, it effectively clears free radicals, promotes local tissue metabolism, stimulates fibroblast proliferation, facilitates collagen fiber formation, and supports angiogenesis. Furthermore, ozone encourages the growth of granulation tissue and epithelial cells (20), thereby aiding tissue repair and positively influencing the healing of DFUs. Ultimately, these beneficial effects of ozone therapy may lead to a shorter inpatient day for patients. In this study, the ozone therapy group also had a shorter inpatient day (18.65 ± 4.93 days) compared to the control group (15.66 ± 4.01 days). In contrast, other studies, such as the one conducted by Rosul et al., reported an inpatient day of 23.42 ± 0.45 days in the control group and 17.09 ± 0.27 days in the ozone therapy group (13). Likewise, Dhamnaskar et al. reported a median average hospitalization time of 13 days versus 9 days (11). These variations in hospitalization time may stem from factors such as the severity of the foot wounds in the selected patients, the concentration of ozone used in treatment, the duration of treatment, and the frequency of treatment.

According to the Bates-Jensen Wound Assessment tool, this study demonstrated a significant improvement in DFUs with ozone therapy, which is consistent with findings reported by Zhang et al. (14), and the study by Kasmawati et al. suggested no significant effect of ozone on wound healing (12), despite the usage of different wound assessment tools. In our study, the ozone therapy group exhibited lower levels of ESR, CRP, and PCT compared to the control group, indicating a potential role of ozone in reducing wound inflammation. These inflammatory markers, ESR, CRP, and PCT, have been previously associated with DFUs prognosis (21–23), aligning with other reports suggesting that ozone therapy can effectively reduce inflammation in DFUs (15, 24, 25).The underlying mechanism for these observations may involve the bactericidal effects of ozone. Ozone has the ability to eliminate bacteria within the wound, consequently reducing damage caused by bacterial colonization to the epithelial cells. This process alleviates wound inflammation and decreases the presence of inflammatory cells and factors in the bloodstream. Ozone therapy has also demonstrated the ability to inhibit the production of cytokines such as IL-6 and TNF-α. Lower levels of these cytokines are beneficial for promoting the healing and repair of wounds (26, 27). Supporting our findings, our study also demonstrated lower levels of IL-6 and TNF-α in the ozone therapy group.

Ozone has the capability to eliminate free radicals, enhance local tissue metabolism, stimulate the division of fibroblast cells, and promote the formation of collagen fibers. It facilitates the secretion of growth factors by macrophages and fibroblast cells, leading to angiogenesis and the growth of granulation tissue, resulting in an accelerated wound healing process. The therapeutic mechanism of ozone in skin wound healing can be attributed to the upregulation of growth factors such as vascular endothelial growth factor (VEGF), transforming growth factor-beta (TGF-β), and platelet-derived growth factor (PDGF) (28, 29). These growth factors play a crucial role in regulating cellular proliferation during tissue repair. By stimulating these growth factors, the regenerative capacity of cells increases, thereby expediting the wound healing process. In our study, the average level of VEGF in the ozone therapy group was found to be higher than that in the control group, indicating its involvement in the formation of granulation tissue (30).

DFUs are characterized by uncontrolled oxidative stress and reduced antioxidant capacity, leading to an imbalance in oxidation-reduction. Excessive oxidative stress can impair all stages of DFUs repair. SOD plays a critical role in the antioxidant process by effectively scavenging harmful reactive ozone species and reducing oxidative stress-induced tissue damage. A study conducted by Gregorio et al. showed that after ozone therapy in DFUs patients, SOD activity increased, potentially due to improved ozone supply, enhanced tissue blood circulation, and activation of the body’s antioxidant defense system (31). T-AOC reflects the overall antioxidant capacity of the body. Following ozone therapy, T-AOC activity significantly increases, thus reducing oxidative stress. MDA is an indicator of oxidative stress levels. Ozone therapy can lower MDA levels, reducing lipid peroxidation reactions, mitigating tissue damage, and promoting DFUs healing. Previous research has also demonstrated ozone’s ability to decrease oxidative stress in conditions such as COVID-19 and lumbar disc-related radicular pain (32, 33).

In our 12-week follow-up, the reinfection rate in the ozone therapy group was 7.3%, compared to 22.9% in the control group, demonstrating the effectiveness of ozone therapy. The control group exhibited a higher readmission rate, likely attributed to the development of new ulcers and reinfections. As the wound healing time extends, the likelihood of new ulcers and reinfections increases. Although our study did not show a reduction in amputation rate with ozone treatment, Dhamnaskar et al. and Izadi et al. reported a decrease in the likelihood of wound reamputation in the ozone therapy group (11, 15), which contrasts with our findings.

This study has several limitations. Firstly, it is a retrospective study, while prospective randomized controlled studies provide stronger evidence. Secondly, the study was conducted at a single center. Lastly, our follow-up period was limited to 12 weeks, and longer-term follow-up would yield greater significance.

In conclusion, this study investigates the short-term effects and long-term prognosis of ozone therapy in DFUs. Ozone therapy reduces inflammation, enhances the expression of growth factors, promotes wound healing, shortens healing time, and improves long-term prognosis. Therefore, advocating for the clinical application of ozone therapy in DFUs management is well-founded.

## Data availability statement

The raw data supporting the conclusions of this article will be made available by the authors, without undue reservation.

## Ethics statement

The studies involving humans were approved by ethics committee of Xuzhou Central Hospital. The studies were conducted in accordance with the local legislation and institutional requirements. The participants provided their written informed consent to participate in this study. Written informed consent was obtained from the individual(s) for the publication of any potentially identifiable images or data included in this article.

## Author contributions

HS: Conceptualization, Writing – original draft. HH: Investigation, Writing – review & editing. XL: Formal Analysis, Writing – review & editing. HG: Conceptualization, Writing – review & editing. JL: Conceptualization, Methodology, Writing – review & editing.
