# Supplementary material for: Evaluation of the healing potential of short-term ozone therapy for the treatment of diabetic foot ulcers
Source: Front Endocrinol (Lausanne). 2024 Jan 15;14:1304034. doi: 10.3389/fendo.2023.1304034 (PMC10825947; doi:10.3389/fendo.2023.1304034)
Supplement: Supplementary file 1 [file Table_1.docx]

| Bacteria isolated | Control | | Ozone | |
| --- | --- | --- | --- | --- |
|  | Pre  (n = 63) | Post  (n = 9) | Pre  (n = 55) | Post  (n = 2) |
| Positive Gram |  | | | |
| Staphylococcus aureus | 8(12.7) | 2(22.2) | 7(12.7) | 1(50.0) |
| Staphylococcus epidermidis | 4(6.3) | 1(11.1) | 3(5.5) | - |
| Staphylococcus lentus | 2(3.2) | - | 2(3.6) | - |
| Staphylococcus haemolyticus | 2(3.2) | - | 1(1.8) | - |
| Enterococcus faecalis | 3(4.8) | - | 2(3.6) | - |
| Streptococcus agalatiae | 2(3.2) | - | 1(1.8) | - |
| Streptococcus constellatus | 1(1.6) | - | 2(3.6) | - |
| Streptococcus oralis |  | - | 1(1.8) | - |
| Negative Gram |  | | | |
| Escherichia coli | 7(11.1) | 1(11.1) | 6(10.9) | - |
| Klebsiella pneumoniae | 6(9.5) | 1(11.1) | 6(10.9) | - |
| Klebsiella oxytoca | 2(3.2) | - | 1(1.8) | - |
| Klebsiella ozaenae | 1(1.6) | - | - | - |
| Klebsiella ornithinolytica | - | - | 1(1.8) | - |
| Morganella morganii | 3(4.8) | - | 2(3.6) | - |
| Enterobacter cloacae | 3(4.8) | - | 2(3.6) | - |
| Proteus mirabilis | 7(11.1) | 1(11.1) | 5(9.1) | - |
| proteus penner | 2(3.2) | - | 1(1.8) | - |
| Citrobacter Koseri | - | - | 1(1.8) | - |
| Citrobacter freundii | 1(1.6) | - | - | - |
| Citrobacter youngae | 1(1.6) | - | 2(3.6) | - |
| Pseudomonas aeruginosa | 6(9.5) | 2(22.2) | 5(9.1) | 1(50.0) |
| pseudomonas stutzeri | - | - | 1(1.8) | - |
| Acinetobacter baumannii | 2(3.2) | 1(11.1) | 3(5.5) | - |

**Supplementary Table 1 Change of Bacterial Types between the two groups**
